# Limiting-Stress-Elimination Hypothesis: Using Non-hormonal Biostimulant to Reduce Stress and Increase Savanna Cowpea [*Vigna unguiculata* (L.) Walp.] Productivity

**DOI:** 10.3389/fpls.2021.732279

**Published:** 2021-08-20

**Authors:** Acheampong Atta-Boateng, Graeme P. Berlyn

**Affiliations:** School of the Environment, Yale University, New Haven, CT, United States

**Keywords:** biostimulant, cowpea, osmotic stress, nitrogen, fertilizer, sprengel-liebig law, sustainable agriculture

## Abstract

An alternative decision axiom to guide in determining the optimal intervention strategy to maximize cowpea production is proposed. According to the decrement from the maximum concept of Mitscherlich, the decrement from the maximum for each stressor must be minimized to produce the absolute maximum production. In crop production, this means all deficient nutrients must be supplemented to ensure maximum yield and laid the foundation in fertilizer formulation. However, its implementation is not economically feasible in many situations, particularly where multiple environmental factors impact crop productivity as in the case of low resource conditions. We propose and test the hypothesis that yield allocation will increase when the most limiting stressor among prevailing stressors is eliminated at least until the next limiting stressor impacts productivity. We selected drought limiting savanna conditions and cowpea (*Vigna unguiculata*), adapted to nitrogen dependence. To determine the limiting condition, we measured the response of cowpea to D-sorbitol, nitrogen, and non-hormonal biostimulant (nhB) treatments. The nhB treatment increased total biomass by 45% compared to nitrogen, 13%, and D-sorbitol, 17%, suggesting osmotic stress is more limiting in the observed savanna conditions. The effect of the biostimulant is due to antioxidants and key amino acids that stimulate metabolism and stress resistance. Where nitrogen becomes the next constraining factor, biostimulants can contribute organic nitrogen. The study supports the use of biostimulants as candidate intervention under conditions where crop productivity is limited by multiple or alternating constraints during crop growth.

## Introduction

The plant stress–response–feedback continuum is an important physiological process that shapes plant plasticity. Factors that account for stress in plants are broadly categorized into abiotic and biotic stressors. Biotic stressors, such as herbivory, pest attack, and disease-causing pathogens, and abiotic stressors, such as drought, salinity, heavy metals, floods, and extreme temperatures, account for major crop losses globally (Gull et al., 2019). To understand how plants respond and cope with stress, a stressor, such as heat or drought, is introduced to the living plant specimen under controlled conditions to observe the response (Arbona et al., 2016; Atta-Boateng et al., 2019). However, under field conditions, crops are exposed to multiple environmental factors (Chapin et al., 1987). In addition, the relative impact of all the possible prevailing stressors on yield is difficult to detect. Furthermore, the intensity of the stress effect does not stay constant throughout the growing season. The centralized system of stress response concept suggests that all plants respond to different stress types in the same basic way, particularly those adapted to low-resourced environments or adjusted to low resource supply (Chapin, 1991). This framework has implications on the implementation of agricultural advances and oversimplifies interventions, especially under marginal conditions where crops, such as legumes, are adopted to low soil nutrients or drought.

Cowpea is the most cultivated legume in West Africa, and its production benefits human and livestock nutrition in addition to long-term soil amendment that sustains cropping systems on marginal lands in low-income economies (Dakora and Keya, 1997; Duranti and Gius, 1997; FAO, 2004; IITA, 2010; Foyer et al., 2016). Cowpea is suggested to originate from West and Central Africa, which currently has most of the germplasm (Boukar et al., 2019b). The biotic constraints of cowpea include parasitic weeds, insect pests particularly aphids, fungal, viral, and bacterial diseases, whereas high temperature, drought, and erratic rainfalls during the growing season constitute major abiotic constraints (Boukar et al., 2019a,b; Horn and Shimelis, 2020). Biotic and abiotic interventions in the past few decades include the introduction of drought-resistant and disease-tolerance cowpea varieties and supporting genetic breeding research programs across Sub-Saharan Africa (SSA) (Boukar et al., 2019a,b). These are essential for managing long-term biotic and abiotic growth conditions. However, major gaps exist for inputs required to manage short-term in-season growth conditions.

Early work by Carl Sprengel and Justus von Liebig's Law of the Minimum revolutionized plant mineral nutrition, where the scarcest nutrient was found to govern growth (Liebig, 1840; van der Ploeg et al., 1999). Later, Mitscherlich (1909), based on Liebig's Law further showed that marginal productivity decreased as levels of limiting growth factor increased. Meaning, when all other factors of growth are kept constant at optimum levels, increase in the levels of the limiting factor increases yield until no further increment affects yield. As all other growth factors cannot be optimal and constant under field conditions, this implies in practice that all stress factors must be eliminated to attain maximum yield. Harmsen (2000) acknowledged the limitations of the Mitscherlich equation in rainfed semi-arid conditions and proposed a modified theory (Equation 1):

(1) $$\begin{array}{r} Y=Y_{\theta }-Y_{\theta }\;e^{-\epsilon_{nt}}N_{t}{Y_{\theta }}^{n-1} \end{array}$$

where *Y* is the total dry matter, *Y*_θ_, potential yield as a function of available moisture (θ), ϵ_*nt*_, activity coefficient, *N*_*t*_, total nutrient content, and *n* is a constant. The model (Equation 1) describes crop response to nutrient availability under rainfed semi-arid conditions and can predict the nutrient requirement for a specified yield level. Nonetheless, the model assumptions restrict practical interventions to mineral nutrient input, given seasonal rainfall availability. The yield impact of limiting factors that alternates during the growing season and the difficulty of its prediction require alternatives frameworks that allow the design of inputs that are pliable to manage dynamic field conditions.

Although SSA contributes more than 70% of global cowpea output, cowpea cultivation is under-produced and concentrated in low-productive regions, which are particularly vulnerable to climate risks (Timko et al., 2008; IITA, 2010; Muñoz-Amatriaín et al., 2017). In the Guinea savanna ecological biome, high temperature, low soil nitrogen, and soil structure exacerbate threats the peak rainy and the dry season conditions cause to agricultural production (Runge-Metzger and Diehl, 1993; Gyasi, 1995). Interventions that can address specific production constraint is currently lacking.

Policy directives on interventions, especially for smallholder farmers in savanna regions continue to focus narrowly on increasing fertilizer inputs (FAO, 2005, 2011; Martey et al., 2014). However, it is unclear whether N fertilizer for instance is the ideal intervention for increasing legume production in the savanna. N fertilization has been shown to decrease N-fixing rates in legumes (McAuliffe et al., 1958; Salvagiotti et al., 2008). Although the application of inoculants in combination with fertilizers on cowpea showed a promising yield effect (Kyei-Boahen et al., 2017), the treatment was beneficial only at specific nutrient deficiency levels. Meanwhile, besides the complexity of the socioeconomic factors that limit fertilizer access in the least productive regions (Erisman et al., 2008; Brown, 2014), the negative environmental impact of misapplied fertilizers is overlooked (Ann and Socolow, 1994; Vitousek et al., 1997). The Ceres2030 report investigated the impact of scientific productivity toward eradicating global hunger (Laborde et al., 2020). The study found that more than 95% of global research interventions toward food security was less relevant toward addressing key need gaps (Laborde et al., 2020). Others have highlighted the need for strategic interventions through sustainable crop production to overcome food insecurity amid climate threats and the rising global population (Tilman et al., 2002; Bita and Gerats, 2013).

In our study, we propose and report a preliminary test of an alternative approach to increase cowpea production under savanna conditions. We hypothesize that productivity, such as growth (total biomass allocation) increases when a relatively more limiting constraint among existing growth constraints is relieved until the next limiting constraint impacts productivity. We formulate a non-hormonal biostimulant (nhB) that treats multiple stressors and tests its effect on nodulation, biomass, and leaf physiological responses in cowpea in comparison with inorganic N fertilizer and exogenous compatible osmolyte.

## Materials and Methods

### Study Area

The study site was located in the Northern region and covers 40% of (92,456 mile^−2^; latitudes 4°-12°N) land cover by area of Ghana. Two major ecological zones characterize the region: the sub-humid to semi-arid Guinea savanna and the arid Sudan savanna zones (Gyasi, 1995). The Inter-Tropical Conversion Zone controls rainfall seasons in Ghana, with one wet season in the north and two wet seasons in the south. Low, erratic rainfall ranging between 150 and 250 mm/month in a single dry season to 1,100–1,200 mm month^−1^ in a single wet season characterizes the study area. Mean monthly temperature during the growing season ranges between 26 and 30°C (Buah and Mwinkaara, 2009). During the study period, the total rainfall (daily mean) recorded was 203.8 (6.6 mm day^−1^); average sunshine was 5.8 h; the mean temperature was 26.7°C (ranging from 29.7°C_max_ to 23.6°C_min_); and the mean relative humidity recorded was 84% (ranging from 93 to 75% min). The soil at the study site constituted 55.8% sand, 41.96% silt, and 2.2% loam. Weather and soil analysis data were obtained from the Savanna Agriculture Research Institute, Council for Scientific and Industrial Research, Ghana.

### Plant Preparation and Treatments

Three experimental blocks, each containing four distinct raised soil beds of dimension 2 × 8 m each and interspaced by a meter gap, were prepared across a slope in a series-like fashion. Each bed in a block represented a treatment group based on a Randomized Complete Block Design. Each soil bed was seeded with cowpea, the common African cultivar Songotra-IT97K-499-35, which is *Striga gesnerioide*s resistant (Asare et al., 2011; Muñoz-Amatriaín et al., 2017).

Germinated seedlings were thinned at 10 cm height to 40 plants for each growing bed of size 80 × 40 cm. Treatments were assigned randomly within and between blocks.

Given the conditions of Guinea savanna, we will use the term osmotic stress to represent the likelihood of both drought and heat stress incidence as they occur in tandem (Dwivedi et al., 2018) or specifically to represent conditions, such as low soil moisture, atmospheric vapor pressure deficit, and salinity, for their similarity in physiological responses (Munns, 2002; Chaves et al., 2009). Three treatments were prepared to treat osmotic stress. First, 0.25 g L^−1^ (coded *sorbL*), and second, 0.5 g L^−1^ (coded *sorbH*) compatible osmolyte, D-Sorbitol (Hexane 1, 2, 3, 4, 5, 6-Hexol) of molecular weight 182.17 g mol^−1^ obtained from Sigma-Aldrich, USA. The third osmotic treatment was an antioxidant and amino acid-based nhB (coded *nhB*), prepared from the laboratory at the Yale School of the Environment. The active components of nhB include 2-amino-5-guanidinopentanoic acid, L-ascorbic acid, thiamine mononitrate, and *cis*-1, 2, 3, 5*-trans*-4, 6-cyclohexanehexol. To arrive at the final treatment formulation, different iterations of nhB beta were evaluated in root-shoot allocation experimental trials in a glasshouse using *Raphinus sativus* (cherry belle variety) as a model plant. The satisfactory formulation was attained when *R. sativus* maintained a fresh root: shoot biomass ratio of ~1:1 and yields over 100%. By this approach, we assumed that the overall yield output of nhB treatment will vary by species type and allocation cost to stress response depending on field conditions.

Furthermore, 5 g L^−1^ nitrogen was prepared from 98% ammonium nitrate (NH_4_NO_3_) as inorganic nitrogen treatment (coded *Nfert*). All control groups were treated with equal volumes of distilled water, which was used to prepare treatment solutions. Each plant replicate received 50 ml of treatment dose biweekly until the onset of pod formation. Nitrogen was applied directly to the soil around the stem of plants while *sorbL, sorbH, nhB*, and controls were applied to both foliage and soil.

### Plant Sampling and Measurement

Out of 40 plant without growth defects, 15 plants were tagged for repeated measurements throughout the growth stages. Leaf measurements were conducted during the active photosynthetic period in the morning (PPDF ranged from 900 to 1,200 mmol m^−2^s^−1^). Leaf temperature, chlorophyll fluorescence, and chlorophyll content of the youngest fully expanded leaf of each tagged plant were measured 2 weeks after each treatment time during the vegetative, flowering, and pod growth stages of cowpea. We used common plant biophysical markers (Ernst, 1999) and traits in our assessment to allow reliable reproducibility and convenience in the study sites.

### Biomass and Nitrogen-Fixing Traits

The root zones were softened by inundating with water to allow the whole plant to be removed from the soil bed with intact roots. The soil debris was brushed off, and the root nodules per plant were carefully removed, counted, weighed, and re-weighed after oven drying to a constant weight at 60°C to obtain the total weight of nodule per plant and the total number of nodules formed per plant. Furthermore, whole plants with intact pods were oven-dried to constant weight to obtain total biomass, then followed by separate measurements of pods.

### Leaf Physiological Measurement

Leaf temperature, *T*_*l*_ was measured as an indicator of plant stress (Udompetaikul et al., 2011; Rodríguez et al., 2015; Carroll et al., 2017). Variations in moisture stresses are known to significantly alter leaf temperature, causing it to deviate from ambient temperature (Wiegand and Namken, 1966). *T*_*l*_ was measured by a laser-guided infrared thermometer (ST60 ProPlus™ Raytec, Santa Cruz, CA, USA). The laser was pointed to the adaxial surface of the leaf, where the device collects optically emitted, reflected, and transmitted energy by the leaf surface on a detector as an ambient temperature reading in °C (±0.07°C).

The stress manifested in plants can be determined by observing the leaf photosystem II (PSII), which is a protein complex in the light-dependent reactions of plant photosynthesis. PSII damage is the first manifestation of stress in plant leaves (Maxwell and Johnson, 2000), and the efficiency of this state is referred to as photochemical efficiency. Maximum quantum yield at PSII is determined based on the chlorophyll fluorescence ratio, $F_{v}{F_{m}}^{-1}$, a well-studied phenomenon in the plant physiology (Rodríguez et al., 2015) and a well-established physiological indicator of plant tolerance to the environmental stress, including drought and heat (Méthy et al., 1994; Maxwell and Johnson, 2000; Dwivedi et al., 2018). Plant stress that affects the PSII is determined based on a calculated variable fluorescence(*F*_*v*_) to maximal fluorescence (*F*_*m*_) ratio using a chlorophyll fluorescence device (model OS-30p, Opti-Sciences, Inc. Hudson, New Hamshire, USA). To measure $F_{v}F_{m}^{-1}$, leaf samples were dark-adapted by placing leaves inside plastic cuvettes to block the incident light on the selected portion of the leaf blade for 30 min. Then a weak modulated light is immediately irradiated from a fluorometer on dark-adapted leaf blade to excite a pre-photosynthetic antenna. Upon light saturation at maximum light exposure, the maximum fluorescence, *F*_*m*_, is determined and $F_{v}F_{m}^{-1}$ computed and recorded by the fluorometer. Chlorophyll fluorescence is one of the most frequently used plant measurement techniques (Schreiber and Bilger, 1993) and has been used extensively in stress studies in several legumes (Georgieva and Yordanov, 1993; Stoddard et al., 2006). The leaf chlorophyll content is a physiological trait associated with drought and heat stress (Dwivedi et al., 2018). A handheld chlorophyll-detecting device, SPAD-502 (Minolta Camera Co., Osaka, Japan), was used to measure the leaf chlorophyll content. The sample leaf was placed in between a receptor window and a measuring head. By pressing the measuring head, the chlorophyll content was determined at 650 and 940 nm in SPAD units at ±1.0 accuracy (see Richardson et al., 2002).

### Statistical Analysis

The Lme4 package in R (Bates et al., 2015) was used to perform mixed-effect modeling (MEM) by restricted maximum likelihood *t*-tests. MEM performs ANOVA on repeatedly measured variables and estimates fixed and random sources of variation. To know where significant differences occurred, we tested general linear hypotheses by multiple pairwise comparisons of means based on Tukey's honestly significant difference (HSD) tests on normally distributed data. All statistical analyses were conducted using the R programming language and environment (R Core Team). The null hypothesis (H_0_) assume no differences in population means such that: *T*_1_ (control), = *T*_2_ = *T*_3_ = … *T*_*x*_; where α ≤ 0.05 and *T*_*x*_ indicates *x* treatment type. Furthermore, the alternative hypothesis (H_a_) assumes differences in population means such that: *T*_1_ ≠ *T*_2_, or *T*_3_, = … *T*_*x*_; at least at a significance level of *p* < 0.05.

## Results

### Effect of Treatments on Biomass, Pod Capacity, and Root Nodulation

The mean biomass of cowpea was significantly different between nhB and all other treatments (*p* < 0.001, Table 1). nhB had the highest biomass output, 45% followed by sorbL (17%), sorbH (13.3%), Nfert (13.1%), and controls. Nodulation capacity determined by the means of the total number of nodules formed per cowpea was significantly different (*p* < 0.01) among treatment groups.

**Table 1:** Summary ANOVA of mean response of treatment groups.

**Parameter variables**		**DF**	***F*-Value**	**(Pr > F)**
Biomass	Treatment	4	6.1889	<0.0001^***^
	Blocks	2	4.907	0.0082^**^
No. of pods	Treatment	4	1.598	0.1759^ns^
	Blocks	2	4.207	0.016^*^
Nodule mass	Treatment	4	0.414	0.798^ns^
	Blocks	2	83.59	<0.0001^***^
No. of nodules	Treatment	4	3.413	0.009^**^
	Blocks	2	55.2	<0.0001^***^

*Superscript stars indicate the levels of significance, where*

* *(0.05)*,

** *(0.01)*,

*** *(0.001), and*

ns *(not significant)*.

**Figure 1:**
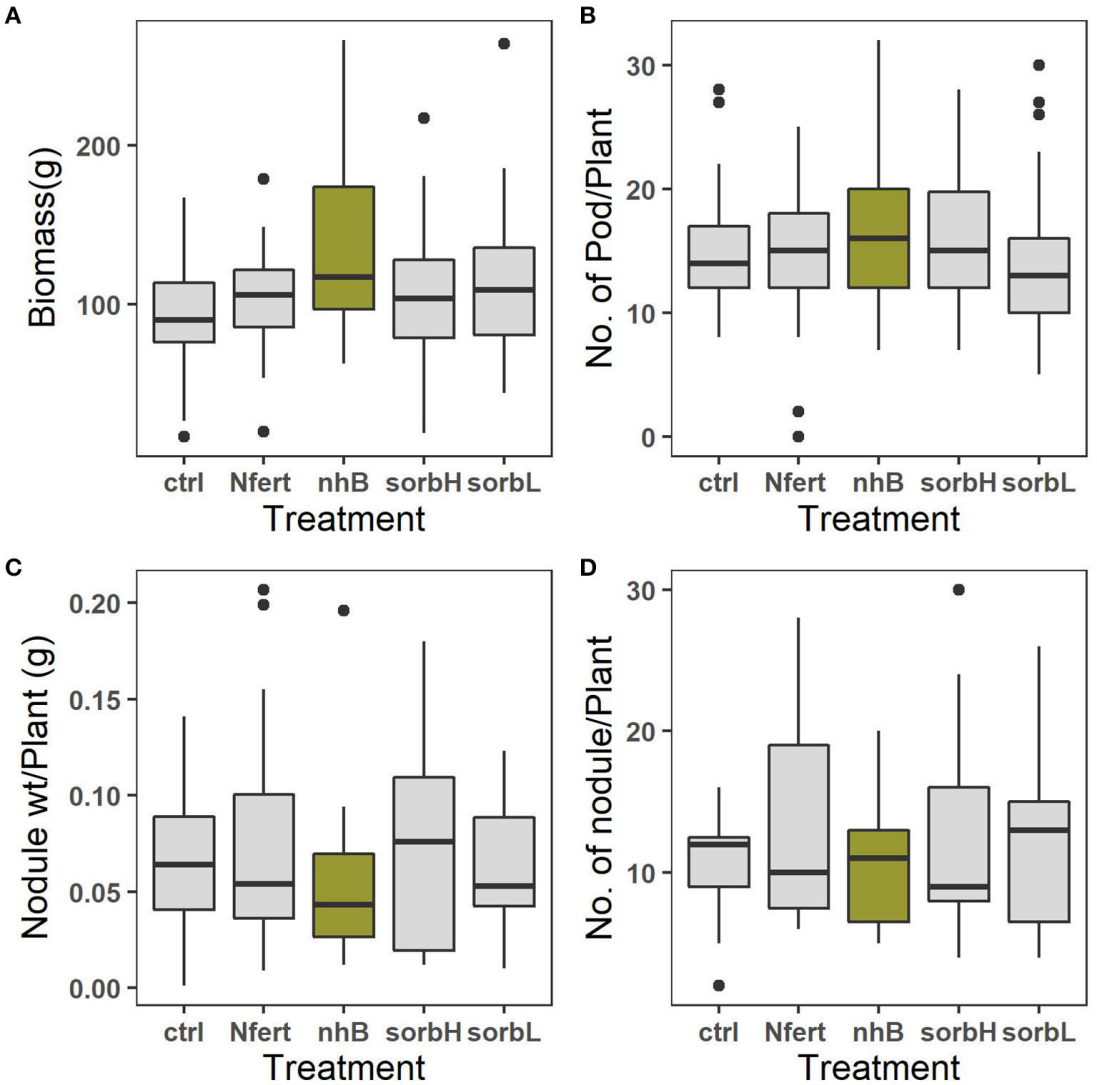
Means of total biomass, grams per plant **(A)**, mean number of pods per plant **(B)**, mean weight of root nodule per plant **(C)**, and mean number of root nodules formed by roots per plant **(D)** in response to cowpea treatments; control, N fertilizer (Nfert), sorbitol (sorbL and sorbH), and non-hormonal biostimulant (nhB).

N-treated cowpea relatively formed the highest number of nodules per root and nodule weight (Figures 1C,D). Thus, we refuse to accept the null hypothesis that there is no difference in the mean effect of treatment interventions on cowpea growth with respect to biomass and nodulation capacity. The effect of blocking was significant on biomass response (*p* < 0.01), number of pods (*p* < 0.05), and mean number and oven-dry weight of nodules (*p* < 0.0001). This indicates that measured responses to treatments are independent of the significant variations in site conditions. This is of statistical importance to emphasize the reliability of the study outcome.

The treatment effects on both the mean number of pods formed per cowpea and the mean nodule mass per cowpea root were not significant (Table 1). Nevertheless, pod yield per plant was relatively highest in nhB-treated cowpea (Figure 1B).

### Effect of Treatments on Leaf Physiological Responses

The mean effect of treatments on leaf chlorophyll content in cowpea did not differ significantly; however, the difference (*p* = 0.05) in leaf chlorophyll responses to sorbL and sorbH (which vary by D-sorbitol concentration) indicates that the concentration of exogenous D-sorbitol application on cowpea leaves might be important to chlorophyll response under savanna conditions. Leaf fluorescence in nitrogen-fertilized cowpea differed significantly from cowpeas treated with nhB, sorbL, and sorbH (Table 2).

**Table 2:** Significant pairs from multiple mean comparisons based on Tukey's honestly significant difference (HSD) test from a linear mixed-effect model fit.

**Parameter variables**	**Treatments**	**Estimate (error)**	***Z*-value**	**(Pr >|z|)**
Chlorophyll fluorescence, FvFm^−1^	Nfert-nhB	0.0368 (0.0128)	2.859	0.033^*^
	Nfert-sorbL	−0.0333 (0.0113)	−2.926	0.028^*^
	Nfert-sorbH	−0.0399 (0.0113)	−3.506	0.004^**^
Chlorophyll content	sorbL-sorbH	−2.5979 (1.0300)	−2.522	0.05
Leaf temperature, *T_*l*_*	Nfert-nhB	−1.1436 (0.2867)	−3.989	<0.001^***^
	Nfert-cntl	−1.3616 (0.2554)	−5.345	<0.001^***^
	Nfert-sorbL	0.7694 (0.2548)	3.020	0.021^*^
	Nfert-sorbH	0.9004 (0.2548)	3.534	0.003^***^

*Except reported, all other possible mean pairs were not significant. Superscript stars indicate the levels of significance, where*

* *(0.05)*,

** *(0.01), and*

*** *(0.001)*.

Treatment effect on leaf temperature, *T*_*l*_ response was significantly different in fertilizer-treated cowpea and the rest of treatment groups (Table 2). Although stress variables are weakly correlated (Supplementary Figure 1), the correlation plot shows the general relation of leaf physiological indicators to aid in the interpretation of leaf treatment responses. Higher leaf temperature does not necessarily indicate a healthy status as in controls. A relatively higher *T*_*l*_, lower leaf chlorophyll content, and fluorescence should indicate a relatively stressful condition.

## Discussion

### Biomass and Physiological Responses of Cowpea to Osmotic and N Treatments

Low soil fertility and drought constitute abiotic factors that constrain cowpea production (Roberts, 2013). Although legumes are adapted to low nitrogen conditions, N-fixation may come at a cost. Nodulation has been used to evaluate stress in legumes (Rai and Singh, 1999). Under savanna conditions, other competing abiotic factors further constrain the biological N-fixing capacity of nodulating legumes and consequently may limit nitrogen contribution to productivity. We hypothesized that where cowpea is constrained by multiple factors, an intervention that alleviates the relatively more limiting factor may lower the physiological cost of mitigating the constraining factor, allowing allocation toward yield. Total biomass and pod yield are used in the study as proxies for productivity or yield allocation. Biomass is a common measure of plant productivity in ecological studies (Roberts et al., 1993; Tilman et al., 1997; Klironomos et al., 2000; van Grunsven et al., 2007; TerHorst and Munguia, 2008; Fraser et al., 2015), and stress evaluation in legume crops (Ashraf and Chishti, 1993; Ashraf and Zafar, 1997; O'Toole et al., 2001). Based on the relatively higher biomass, pod yield, and lower investment in the root nodules in nhB, sorbL, and sorbH cowpea treatments (Figures 1A–D), we conjecture that osmotic stress is more limiting than soil nitrogen need in cowpea in the study area. The additional higher yield in nhB may be due to the combined effect of antioxidants and amino acids used in the formulation. More nodules were formed in untreated controls than biostimulant, nitrogen, and sorbitol treatments and may indicate the reduced need for nitrogen.

**Figure 2:**
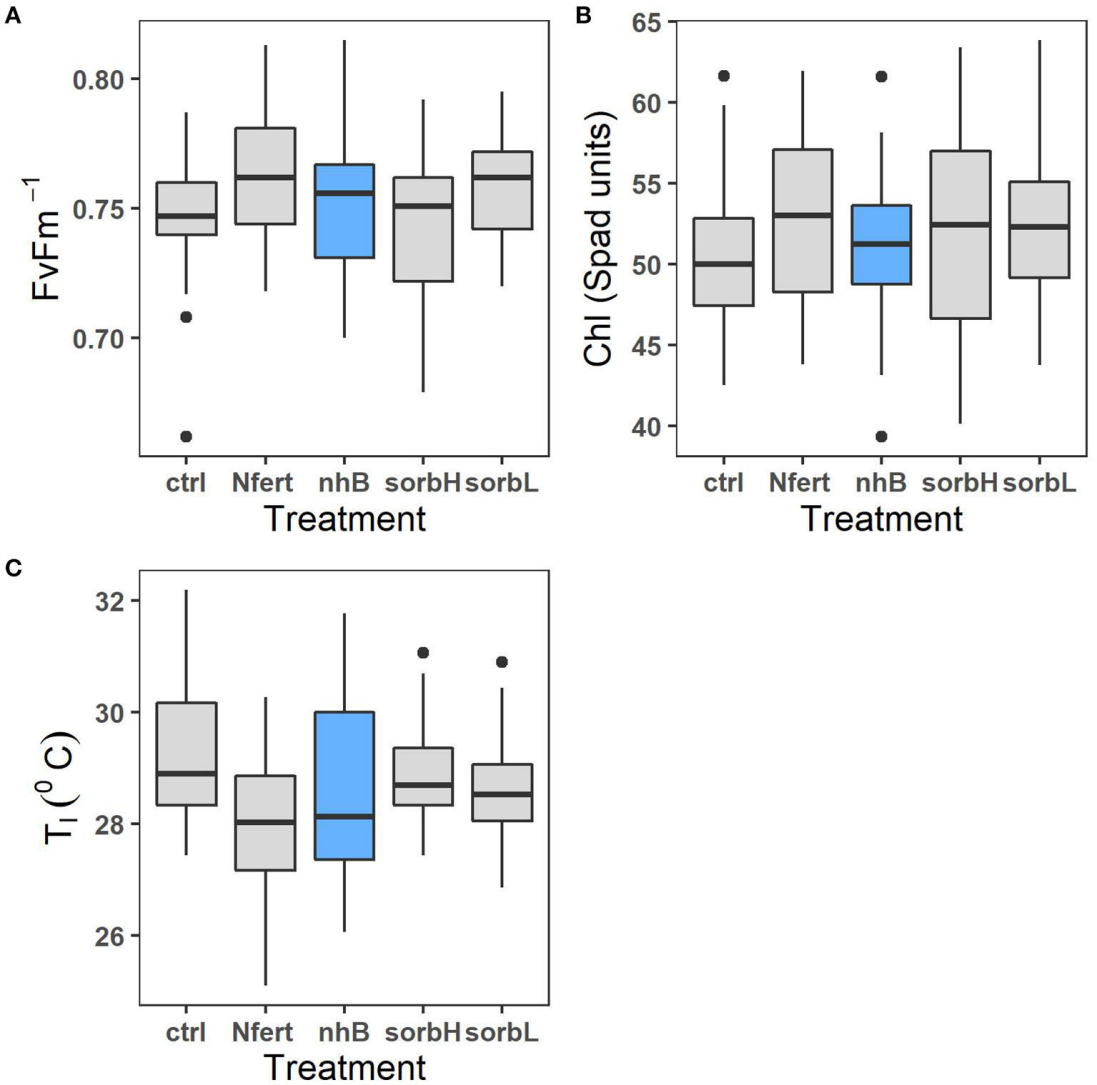
Leaf physiological response of cowpea to osmotic treatments (sorbL, sorbH, and nhB) and N fertilizer treatment (Nfert). Plots represent chlorophyll fluorescence ratio **(A)**, leaf chlorophyll content **(B)**, and leaf temperature **(C)**.

Nitrogen, biostimulant, and sorbitol treatments reduced cowpea stress in contrast with controls (Figure 2). While we expected higher N-fixing capacity and $F_{v}F_{m}^{-1}$ to increase productivity, the response was relatively lower in N treatment than in biostimulant and sorbitol treatments. Maintaining leaf moisture while fixing carbon may be the more efficient strategic role of the osmoprotection compared to N-fertilized cowpea, which showed higher proxy-photosynthetic activity (Figures 2A,B), yet seemingly more leaf water loss, resulting from a cooling effect and lower leaf temperature. The relatively lower biomass and increased nodulation response to N treatment suggest a plausible trade-off to increased investment toward a need for physiological response and N-fixation (Figures 1C,D). Contrary, higher biomass and leaf stress responses to osmotic treatments support the conjecture that exogenous osmolytes alleviate the need and the cost of metabolic response, thereby directing more allocation toward whole-plant biomass output.

### Effect of Non-hormonal Biostimulant and D-Sorbitol on Cowpea

Biostimulants, since their origination, definition, and development over the past four decades, have been shown to improve stress resistance and promote plant development (Russo and Berlyn, 1991). Although drought avoidance by hydraulic controls (Hall and Schulze, 1980; Turk and Hall, 1980) and leaf paraheliotropism (Schakel and Hall, 1979) (also observed in this study) persists in cowpea, osmoadaptation through metabolite accumulation is reported as a more conservative strategy in cowpea (Goufo et al., 2017). The relative increase in biomass, following sorbitol and biostimulant treatments supports osmoadaptation as an important drought response strategy in cowpea. From our results, we conjecture that external application of osmolyte-containing compounds may prevent the inherent metabolic need and cost for cowpeas to initiate osmoadaptation, thereby making readily available more resources for biomass allocation.

In a study of 88 cowpea metabolites, *myo*inositol and arginine were reported to have no beneficial effect on cowpea yield (Goufo et al., 2017). Contrary to our findings, *myo*inositol and arginine, both active constituents of the nhB formulation improved biomass allocation in cowpea. The contrasting findings may result from differences between the metabolite concentration in naturally occurring cowpea leaves and the concentrations used in the nhB formula. Further, our study reports the first yield effects of exogenous D-sorbitol on savanna cowpea. Although D-sorbitol does not constitute the reported metabolites expressed in drought-stressed cowpea, its effect on yield in our study suggests that metabolite–yield relations may be non-species specific. In that, the metabolites expressed by plants under stressful conditions may not represent the only possible strategy but a choice, perhaps governed by adoptive mechanisms peculiar to the species or based on inherent biochemical resources available to initiate the metabolic response process.

### Implication of the Limiting-Stress-Elimination Hypothesis in Savanna Legume Production

**Figure 3:**
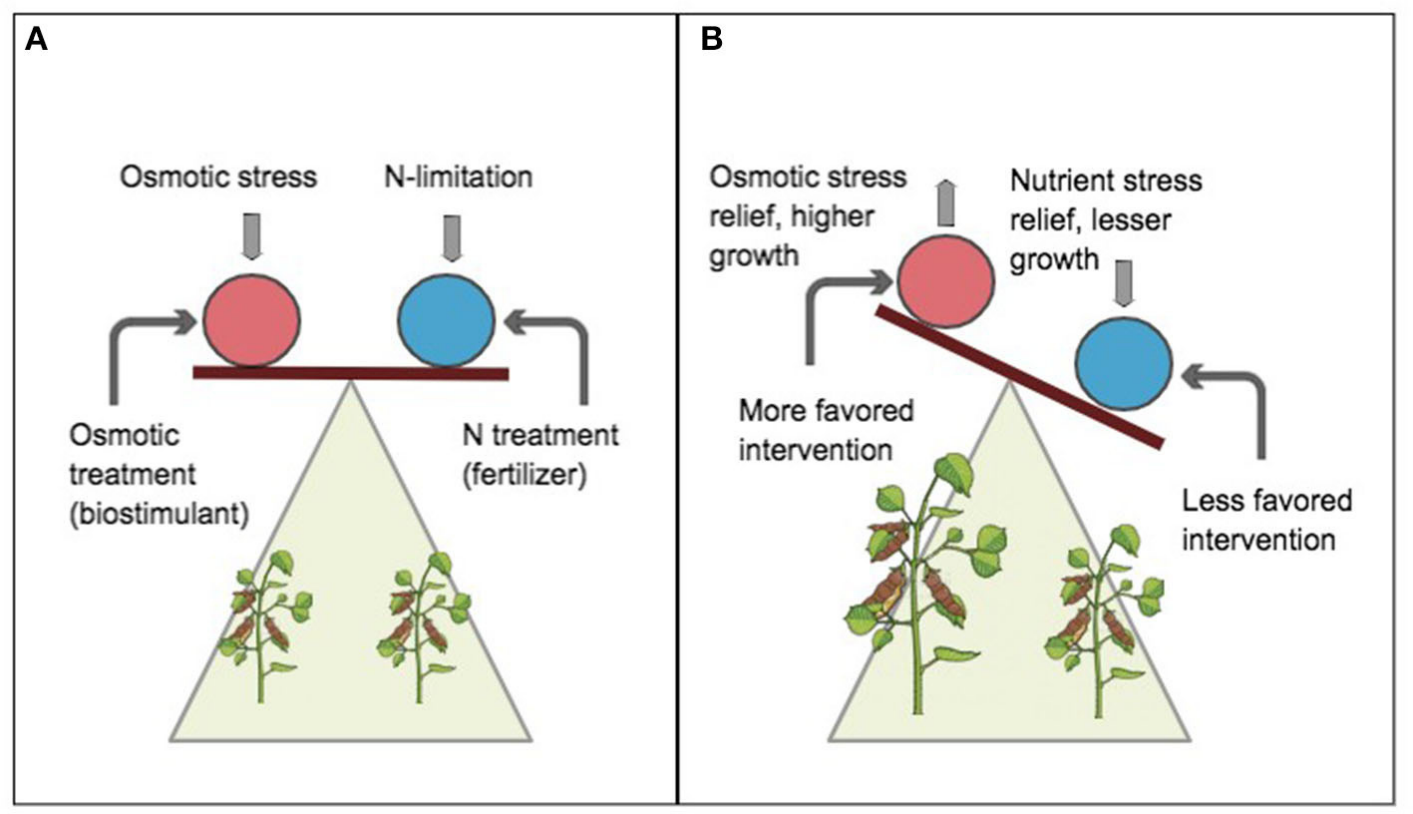
A schematic of the limiting-stress-elimination hypothesis (LSEH). Circles represent whole-plant organisms. Balanced plane implies a notion of significance or pedigree of stress impact. Vertical arrows indicate stressors (osmotic and nitrogen limitation), downward arrow implies stress impact, and upward arrow implies relived stress. Curved arrows indicate applied intervention (exogenous biostimulant and nitrogen deposition). At equilibrium **(A)**, the stressor impact on productivity is assumed similar. Without any intervention, the impact of multiple stressors can be assumed to be synergetic and difficult to detect. However, when defined interventions are simultaneously applied, a shift in slope or tilt **(B)** distinguishes the relative magnitude of the impact of a conjected stressor and how much it limits plant productivity. The favored intervention **(B)** in the red circle shows sensitivity to stress response by plant and eliminates or alleviates the impact of the stressor. This results in increased productivity indicated by an upward tilt (analogous to a “lighter weighing effect”).

According to the proposed limiting-stress-elimination hypothesis (LSEH), when the limiting stress is eliminated, stress impact is relieved (Figures 3A,B), until the next available stress becomes most limiting. The scope of this study here is not to establish the most limiting stressor in cowpea.

Instead, we have demonstrated which, among identifiable constraints relatively limits cowpea productivity most by showing whose “elimination” (by treatment) relatively increased cowpea biomass. This makes the study application relevant to ongoing field situations where stressors may be short-lived yet damaging to yield. Contrary to the law of minimum and diminishing soil productivity (Mitscherlich, 1909) whereby maximum productivity requires relieving all decrements from the optimal level for each nutrient, intervention by the LSEH focuses on the predominant limiting stressor given prevailing conditions and not a host of stressors. In the Guinea savanna of West Africa, where leguminous crops are predominantly constrained by limited soil nitrogen and osmotic stress, the simultaneous treatment to eliminate multiple abiotic stressors may increase farming costs beyond economic practicality.

Increasing legume production constitutes a long-term food security initiative and ecological restoration strategy whereby legumes improve soil N, increase the availability of percent arable lands, and the possible recruitment or cultivation of less competitive but essential non-legume plants or crop species in the savanna. In our study, we measured N-fixation indirectly by the number of nodules formed per plant and dry mass. While N treatment relatively increased nodulation capacity significantly, the effect of inorganic N, the biostimulant, and sorbitol on mean nodule dry mass did not vary significantly. Thus, exogenous osmotic stress treatment constitutes a promising alternative for increasing cowpea production without impairing their biological N-fixing capacity. N-fixation may be an important agroecological tool for improving long-term savanna ecosystem productivity. Where necessary, the use of N fertilizers to maximize legume yield should depend on whether additional inorganic N supplement addresses the yield gap in the specific growing area. Evidence exists to question the relevance of N fertilization in cowpea. For instance, in a study by Martins et al. (2003), bacteria inoculated cowpea improved grain productivity more than N-fertilized cowpea through increased biological N-fixation. In this instance, regardless of N supplement, increased biological N-fixation through bacterial inoculation accounted for higher yield. Essentially, the metabolic cost of microbial-aided N-fixation may be lower than the metabolic cost of converting inorganic N fertilizers to readily available forms to impact yield. Similarly, in our study, minimizing the cost of stress allocation may have enhanced yield in which amino acid in nhB contributes organic nitrogen. Besides direct plant growth, biostimulants can be designed to supplement the resource needs of N-fixing bacteria to indirectly improve yield.

It can be inferred from biomass responses that a less productive outcome may ensue when an intervention targets a lesser limiting factor, while the relatively more limiting factor persists, as in the common practice of fertilizer use in crop production in arid landscapes. Hence, it becomes imperative for the agronomist to determine what constitutes the measurable limiting factor for a crop, given its growth conditions. When that is determined, management techniques and tools that improve resource use efficiency become the next relevant agronomic asset (Chaves et al., 2009). The LSEH utilizes a cost–benefit utility that should inform decisions and potential innovations entrenched in the principle of efficiency and sustainability in agricultural practices, particularly in regions challenged by both limited economic and environmental constraints. In the search for sustainable agroecological solutions, LSEH can assist agronomists to decipher appropriate interventions optimal for improving productivity.

The yield effect of biostimulant on N-fixing crops and trees and as a low input for sustainable agriculture has been discussed (Berlyn and Russo, 1990; Russo and Berlyn, 1991, 1992). The goal of this study was to demonstrate the potential of nhB as an input specifically in conditions where fluctuating diurnal or within-season temporal biophysical stressors can impact yield. Although the study satisfies statistical assumptions, it is a test of hypothesis limited to a single species as such, further inquiry can be useful to expand the scope and limitations of the hypothesis. However, from years of experience in biostimulant formulation, there is a remarkable advantage, especially when adopting core formula to other crops and often require small adjustment in the constituent compounds and or their concentrations. Further, our study does not report on the potential implementation cost. Earlier developments, including ROOT 1™ and ROOT 2™ that constituted mycorrhiza were successfully commercialized and available at low cost, and we assume that recent developments based on more available resources will likewise make nhB accessible at low cost.

## Conclusions

Crops growing in the Guinea savanna sub-biome are constrained by a myriad of factors including low soil N, high temperature, and drought. Eliminating each constraint as proposed by the decrement from the maximum concept can be impractical, especially in low-income economies that depend on rainfed agriculture. Although fertilizer input remains the predominant approach to crop nutrient enrichment, it is beneficial where soil nutrient deficit mostly limits crop productivity. The study hypothesized that productivity is relatively maximized when a relatively more limiting stress factor to growth is relieved among other possible stressors. In a case study with cowpea growing in the savanna conditions, osmotic stress treatment increased yield more than N treatment. However, non-hormonal biostimulant treatment, which constitutes both osmolyte and organic nitrogen sources impacted yield most. We conjecture that biostimulants can better manage crop production where productivity may be limited by alternating factors across the growing cycle or by multiple constraints.

## Data Availability Statement

The original contributions presented in the study are included in the article/Supplementary Materials, further inquiries can be directed to the corresponding author/s.

## Author Contributions

AA-B and GB conceived the idea, interpreted the results, and wrote the manuscript. AA-B conducted experiments, collected data, and performed the statistical analysis.

## Conflict of Interest

The authors declare that the research was conducted in the absence of any commercial or financial relationships that could be construed as a potential conflict of interest.

## Publisher's Note

All claims expressed in this article are solely those of the authors and do not necessarily represent those of their affiliated organizations, or those of the publisher, the editors and the reviewers. Any product that may be evaluated in this article, or claim that may be made by its manufacturer, is not guaranteed or endorsed by the publisher.
