# Supplementary material for: Limiting-Stress-Elimination Hypothesis: Using Non-hormonal Biostimulant to Reduce Stress and Increase Savanna Cowpea [Vigna unguiculata (L.) Walp.] Productivity
Source: Front Plant Sci. 2021 Aug 20;12:732279. doi: 10.3389/fpls.2021.732279 (PMC8417892; doi:10.3389/fpls.2021.732279)
Supplement: Supplementary file 1 [file Data_Sheet_1.docx]

**Supplementary**

Sup 1. Correlation of stress indicator variables. A) Leaf temperature (Leaf Temp ^0^C) and $F_{v}F_{m}^{-1}$. B) Leaf temperature (Leaf Temp ^0^C) and Chlorophyll content. C) Chlorophyll content and $F_{v}F_{m}^{-1}$.
